# Paraptosis triggers mitochondrial pathway-mediated apoptosis in Alzheimer’s disease

**DOI:** 10.3892/etm.2021.10715

**Published:** 2021-09-09

**Authors:** Dong-Pei Jia, Song Wang, Bao-Chao Zhang, Fang Fang

Exp Ther Med 10:804–808, 2015; DOI: 10.3892/etm.2015.2531

Following the publication of the above paper, a concerned reader drew to the Editor’s attention that a pair of panels in Fig. 1B had been inserted into Fig. 1 twice in different orientations or at a different brightness setting, where the data were intended to have shown the results obtained under different experimental conditions; furthermore, Fig. 2 also appeared to show evidence of repetitive features in terms of the layout of cells, and arrangements of the cells, within or between panels.

After having conducted an independent investigation in the Editorial Office, the Editor of *Experimental and Therapeutic Medicine* has determined that this article should be retracted from the Journal on account of a lack of confidence in the authenticity of the data. The authors were asked for an explanation to account for these concerns, but the Editorial Office never received any reply. The Editor regrets any inconvenience that has been caused to the readership of the Journal.

